# Bedeutung der Desorientierung bei der Delireinschätzung: Sekundäranalyse einer prospektiven, observationellen Studie

**DOI:** 10.1007/s00063-021-00850-z

**Published:** 2021-08-25

**Authors:** Ulf Guenther, Mirko Wolke, Hans-Christian Hansen, Nicole Feldmann, Anja Diers, Oliver Dewald, E. Wesley Ely, Andreas Weyland

**Affiliations:** 1 Universitätsklinik für Anästhesiologie/Intensivmedizin/Notfallmedizin/Schmerztherapie, Klinikum Oldenburg AöR, Universitätsmedizin Oldenburg, Rahel-Straus-Str. 10, 26133 Oldenburg, Deutschland; 2 Fakultät VI – Medizin und Gesundheitswissenschaften, Carl von Ossietzky Universität, Oldenburg, Deutschland; 3 Klinik für Neurologie, Friedrich-Ebert-Krankenhaus Neumünster GmbH, Neumünster, Deutschland; 4 Universitätsklinik für Herzchirurgie, Klinikum Oldenburg AöR, Oldenburg, Deutschland; 5 Critical Illness, Brain Dysfunction, and Survivorship (CIBS) Center, Vanderbilt University Medical Center, Nashville, TN USA; 6 Department of Medicine, Division of Pulmonary and Critical Care Medicine, Vanderbilt University School of Medicine, Nashville, TN USA; 7 Geriatric Research Education Clinical Center (GRECC), Department of Veterans Affairs, Tennessee Valley Healthcare System, Nashville, TN USA

**Keywords:** Postoperativ, Intensivstation, Intermediate Care, Herzchirurgie, Orientierungsstörung, Postoperative, Confusion, Intensive care unit, Intermediate care, Cardiac surgery

## Abstract

Desorientierung kann ein frühes Merkmal eines Delirs sein. Für die Überwachung eines Delirs testet die im deutschsprachigen Raum weit verbreitete „Confusion Assessment Method for Intensive Care Unit“ (CAM-ICU) die Orientierung nicht, da intubierte Intensivpatienten sich nicht verbal äußern können. Die Mehrheit der Patienten auf deutschen Intensivstationen ist aber nicht beatmet, sie könnten hinsichtlich ihrer Orientiertheit befragt werden. Die vorliegende Studie untersuchte, ob sich durch das Kriterium „Desorientierung“ bei extubierten Patienten im Vergleich zur CAM-ICU divergierende Befunde ergeben und ob sich die Sensitivität der CAM-ICU durch Kombination mit dem Merkmal „Desorientierung“ („CAM-IMC“) erhöhen lassen. Insgesamt 86 gepaarte Untersuchungen fanden bei 50 extubierten Patienten statt. Ein Delir fand sich bei 19,8 % (*n* = 17) aller Untersuchungen. Die CAM-ICU hatte eine Sensitivität von 71 % (95%-KI: 44–90 %) und eine Spezifität von 100 % (95–100 %). Für „Desorientierung“ als alleiniges Delir-Merkmal fand sich eine Sensitivität von 77 % (50–93 %) und eine Spezifität von 93 % (89–100 %). Die CAM-IMC erreichte eine Sensitivität von 88 % (64–99 %) bei einer Spezifität von 100 % (95–100 %). Die „Receiver-Operating-Characteristics(ROC)-Analyse“ fand mit einer „area under the curve“ (AUC) von 0,941 (95%-KI: 0,851–1,000) für die CAM-IMC den höchsten Wert im Vergleich zu den anderen Delir-Tests (CAM-ICU, AUC 0,853 [0,720–0,986]; Desorientierung, AUC 0,868 [0,745–0,991]). Diese Arbeit unterstreicht die Wertigkeit des Merkmals „Desorientierung“ für Delir-Tests bei verbal kommunikationsfähigen Patienten und erklärt einige diskrepante Beurteilungen schwierig einzuschätzender Patienten in der täglichen Praxis. Die CAM-IMC scheint als Delir-Test für extubierte Patienten günstigere Eigenschaften als die CAM-ICU zu haben und sollte eingehender überprüft werden.

## Abstract (en)

Disorientation may present as a warning sign of developing delirium. The most commonly used delirium assessment tool in Germany, the Confusion Assessment Method for Intensive Care Unit (CAM-ICU), does not rate “disorientation”, since intubated patients cannot communicate verbally. However, the majority of German ICU patients are not orally intubated, so they could be examined for their orientation. This study was carried out to investigate whether the delirium feature “disorientation” in extubated patients yields diverging findings in comparison to the CAM-ICU and whether the sensitivity of the CAM-ICU may be improved when combined with the feature “disorientation” (CAM-IMC). A total of 86 paired assessments were completed in 50 extubated patients. Delirium was found in 19.8% (*N* = 17). The CAM-ICU had a sensitivity of 71% (95% confidence interval [CI] 44–90%) and a specificity of 100% (95–100%). Disorientation, if taken as the only delirium feature, had a sensitivity of 77% (50–93%) and a specificity of 93% (89–100%). The CAM-IMC reached a sensitivity of 88% (64–99%) and a specificity of 100% (95–100%). The receiver operating characteristics (ROC) analyses found an area under the curve (AUC) of 0.941 (95%CI 0.851–1.000) for the CAM-IMC, which was the highest compared to the other delirium tests (CAM-ICU, AUC 0.853 [0.720–0.986]; disorientation, AUC 0.868 [0.745–0.991]). This research emphasizes the importance of the feature “disorientation” for delirium assessments in patients able to verbally communicate and explains some controversial delirium ratings in daily practice. The CAM-IMC appears to be an attractive tool for delirium assessment in nonintubated patients and deserves further research.

## Hintergrund und Fragestellung

Ein Delir nach Operationen und interventionellen Eingriffen ist eine akute, lebensbedrohliche Organdysfunktion. Gemäß ICD-10 ist es gekennzeichnet durch „gleichzeitig bestehende Störungen des Bewusstseins einerseits und mindestens zwei der nachfolgend genannten Störungen andererseits: Störungen der Aufmerksamkeit, der Wahrnehmung, des Denkens, des Gedächtnisses, der Psychomotorik, der Emotionalität oder des Schlaf-Wach-Rhythmus.“ (ICD-10-GM-2021). Es gilt, ein Delir frühzeitig zu erfassen, da es mit erhöhter Mortalität, verlängertem Aufenthalt auf Intensivstation und im Krankenhaus assoziiert ist [8, 10]. Ein regelmäßiges Delir-Monitoring ist mit einer niedrigeren Mortalität, die verzögerte Therapie eines Delirs mit einer höheren Mortalität assoziiert [18, 24]. Bei Überlebenden kann es zu einer erheblichen Einschränkung der Kognition und Alltagsaktivität kommen [29, 30].

Für die Überwachung eines Delirs empfiehlt die deutschsprachige S3-Leitlinie „Delirmanagement, Analgesie und Sedierung“ [7] neben der „Intensive Care Delirium Screening Checklist“ (ICDSC) [3] die „Confusion Assessment Method for Intensive Care Unit“ (CAM-ICU) [9]. Die CAM-ICU ist im deutschsprachigen Raum der am weitesten verbreitete Delir-Test [20, 31]. Da intubierte Intensivpatienten sich nicht verbal äußern können, wurde die CAM-ICU so konzipiert, dass Patienten sich auf einer „Ja/Nein-Basis“ (z. B. Händedruck, Kopfschütteln) äußern können. Aus diesem Grund musste sie auf die Überprüfung der Orientiertheit verzichten, da sich Patienten hierzu verbal äußern müssten. Zu beachten ist, dass die Orientierungsstörung oder Desorientierung im gegenwärtigen ICD-10-Code des Delirs (ICD-10 F05) nicht vorkommt (s. oben).

Die Orientierung im Raum, in der Zeit und zur Situation ist eine Unterfunktion des Bewusstseins von sich und der Umgebung. Sie ständig aufrechtzuerhalten („context-updating“) erfordert neben Wachheit und Wahrnehmungsschärfe einige intakte Aufmerksamkeits- und vor allem Gedächtnisfunktionen, mithin eine funktionelle Konnektivität beider Hemisphären sowie deren Interaktion mit dem Hirnstamm [17]. Desorientierung als typisches Merkmal des Korsakow-Syndroms („amnestic syndrome“, ICD-10 F04) kann chronisch nahezu ohne bedeutsame Aufmerksamkeitsdefizite vorliegen, also auch ohne typisch delirante Elemente. In diesen Fällen ist das episodische Langzeitgedächtnis so stark beeinträchtigt, dass das zeitliche, räumliche und kausale „biografische Gitter“ nicht mehr abgespeichert bzw. abgerufen werden kann. Dies betrifft Fragen wie „Woher?“, „Wohin?“, „Wann?“ und „Warum?“. Insofern bietet sich die Prüfung der Orientierung als ein Produkt multipler psychischer Leistungen mit Schwerpunkt auf den Gedächtnisfunktionen als ein griffiges Indikatormerkmal der qualitativen Bewusstseinsstörung an.

In Deutschland sind oft nur 20 % bis 50 % der Patienten auf Intensivstation beatmet, könnten hinsichtlich ihrer Orientiertheit also befragt werden [27]. Gelegentlich kommt es aber vor, dass es zu Differenzen zwischen den Ergebnissen validierter Delir-Tests und dem klinischen Eindruck erfahrener Mitarbeiter kommt [13]. Patienten, die gemäß klinischem Eindruck desorientiert sind, erfüllen notwendige Delir-Testkriterien nicht, andere Patienten wirken delirant, sind hingegen aber voll orientiert.

Zum Thema Desorientierheit bei postoperativem Delir liegen kaum Studien vor. Einer systematischen Übersichtsarbeit ist zu entnehmen, dass die Orientierungsstörung eine hohe Sensitivität (90–97 %) bei gleichzeitig niedriger Spezifität für das Vorliegen eines Delirs hat (55–80 %) [2]. Nur eine einzige Arbeit ist in PubMed gelistet, die „disorientation“ und „delirium“ im Titel trägt [21]. Sie beschreibt, dass der Verlust der Orientiertheit ein Frühsymptom eines beginnenden Delirs ist und dass die drei Qualitäten Zeit, Ort und Person nicht gleich vulnerabel sind. Die Qualität „Zeit“ sei die erste Qualität, die leide. Man könne sagen, dass sich der Patient in einem teilweisen Delir befinde [21]. Zur Klärung des Zusammenhangs von Desorientierung und Delir werteten wir daher einen Datensatz aus, der im Rahmen einer prospektiven Delir-Studie erhoben worden war und Delir und Desorientierung gezielt untersucht hatte [14].

Ziel der vorliegenden Studie war es, herauszufinden, ob sich durch das Kriterium „Desorientierung“ bei extubierten Patienten im Vergleich zur Confusion Assessment Method for Intensive Care Units (CAM-ICU) hinsichtlich der Diagnose eines Delirs erhebliche divergierende Befunde ergeben. Ferner wurde untersucht, ob sich Sensitivität und Spezifität der CAM-ICU durch Kombination mit der „Desorientierung“ („CAM-IMC“) erhöhen lassen.

## Studiendesign und Untersuchungsmethoden

Diese Arbeit ist eine Sekundäranalyse einer prospektiven Observationsstudie bei herzchirurgischen Patienten, die postoperativ eines Intensivaufenthalts bedurften [14]. Sie wurde in Übereinstimmung mit der Deklaration von Helsinki und nach Zustimmung durch die zuständige Ethikkommission durchgeführt (Ethikkommission der Universität Oldenburg, Vorsitzender: Prof. Dr. F. Griesinger, Nr. 2019-001, 28. Feb. 2019).

### Patientenkollektiv

Es wurden 52 Patienten/-innen rekrutiert, die sich einer elektiven herzchirurgischen Operation unterzogen. Einschlusskriterien waren Alter > 60 Jahre, wichtigste Ausschlusskriterien waren Ablehnung der Studie und die Unfähigkeit einer präoperativen kognitiven Testung, z. B. durch Fremdsprachlichkeit, bekannte kognitive Einschränkungen und schwere neurologische Störungen. Nach Aufklärung und Einwilligung wurde alle Patienten mit dem Mini-Cog-Test hinsichtlich des Vorliegens einer kognitiven Einschränkung untersucht [4]. Ferner erfolgte eine umfangreiche medizinische Anamnese inklusive der Erhebung der Vormedikation. Postoperativ wurden die Patienten am ersten und vierten bis sechsten postoperativen Tag hinsichtlich eines Delirs überprüft.

### Delir-Monitoring

Präoperative Untersuchungen und postoperative Delir-Tests wurden durch einen speziell geschulten Medizinstudenten durchgeführt (Studienuntersucher) sowie durch einen in der Delirdiagnostik erfahrenen Untersucher vorgenommen (Referenzuntersucher). Hierzu wurden die Patienten unabhängig voneinander gesehen. Der Referenzuntersucher konnte dafür zusätzlich auf die Dokumentation von Pflegepersonal und Ärzten zurückgreifen, welche vorher wiederholt im Delir-Monitoring geschult worden waren. Der Studienuntersucher kannte weder deren Einschätzungen noch die des Referenzuntersuchers.

#### CAM-ICU

Am ersten und fünften postoperativen Tag erfolgte ein Delir-Monitoring mit der CAM-ICU gemäß der S3-Leitlinie [7]. Die CAM-ICU testet positiv für ein Delir, wenn die Merkmale „akuter Beginn oder schwankender Verlauf einer psychischen Veränderung“, „Aufmerksamkeitsstörung“ sowie entweder eine Veränderung der Vigilanz oder eine Denkstörung vorliegt. Auf eine Überprüfung der Orientiertheit muss die CAM-ICU wie oben erläutert verzichten, da intubierte Patienten sich nicht verbal äußern können.

#### 4AT mit „ANANASTEST“

Bei extubierten Patienten wurde zur Erfassung des Delirs der 4AT-Test herangezogen [2]. Für die Delir-Einschätzung mithilfe des 4AT wurde in dieser Studie für das Merkmal „Aufmerksamkeitsstörung“ nicht, wie im Original, der „Monate-Rückwärts-Test“ („months of the year backwards“, MOTYB), sondern der Vigilance-„A“-Test („ANANASBAUM“) verwendet [15]. Dies geschah, weil Letzterer auf deutschen Intensivstationen gut etabliert ist [20, 33]. Ein wesentlicher Unterschied des 4AT zur CAM-ICU ist die Überprüfung der Desorientierung. Hierfür testet der 4AT vier Kategorien der Orientiertheit ab: Geburtsdatum, Alter, Ort und aktuelles Jahr [36].

#### Confusion Assessment Method für nichtintubierte Patienten (CAM-IMC)

Zur Überprüfung, ob durch die Kombination der CAM-ICU mit dem Merkmal „Desorientierung“ vorteilhaftere Testgütekriterien zu erreichen sind, wurde die CAM-IMC konstruiert. Hierfür wurde das Merkmal „Denkstörung“ durch die „Orientierungsstörung“ des 4AT ersetzt. Beim 4AT wird ein Fehler mit einem Punkt, mehr als ein Fehler mit 2 Punkten gewertet. Für die CAM-IMC wurde – in Analogie zum Merkmal „Denkstörung“ der CAM-ICU – die Störung nur einer Qualität der „Orientierung“ nicht als Fehler gewertet, zwei Störungen hingegen als Fehler.

**Figure 1:**
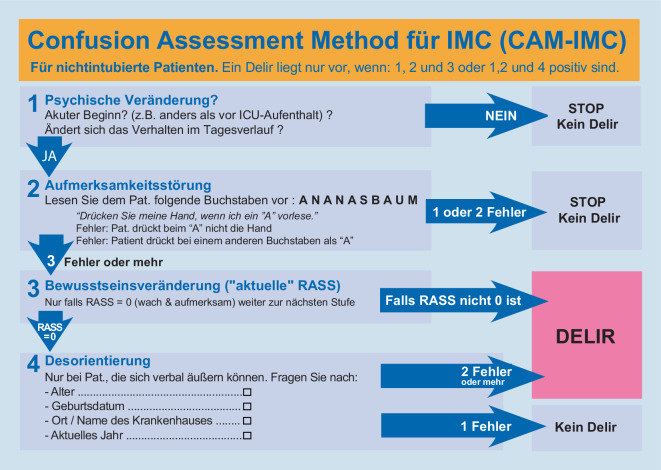

Die Abb. 1 verdeutlicht den Algorithmus der CAM-IMC. Analog zur CAM-ICU testet sie vier Merkmale des Delirs nacheinander ab: Nach Merkmal 1 (psychische Veränderung – akuter Beginn oder schwankender Verlauf?) wird die Aufmerksamkeitsstörung überprüft. Hierzu wird die Buchstabenfolge „A N A N A S B A U M“ langsam vorgelesen. Die Patienten sollen jeweils die Hand der Untersucher nur drücken, wenn ein „A“ vorgelesen wird. Alles andere wird als Fehler gewertet. Werden hier maximal zwei Fehler gemacht, so liegt laut Algorithmus kein Delir vor. Bei mehr als zwei Fehlern erfolgt die Erhebung der Bewusstseinsveränderung durch Anwendung der Richmond Agitation-Sedation Scale (RASS). Ist diese größer oder kleiner Null (RASS ≠ 0), liegt – bei gleichzeitigem Vorliegen einer akuten Aufmerksamkeitsstörung (Merkmale 1 und 2) – ein Delir vor (s. auch „Vigilanz und Analgesie“). Wirken bei Vorliegen einer akuten Aufmerksamkeitsstörung (Merkmale 1 und 2) Patienten äußerlich aufmerksam und ruhig (RASS = 0), so muss die Orientierungsstörung getestet werden (Merkmal 4 der CAM-IMC). Bei nur einem Fehler wurde für diese Arbeit das Merkmal als negativ gewertet, bei zwei oder mehr Fehlern wurde eine Orientierungsstörung angenommen.

### Vigilanz und Analgesie

Sediertheit bzw. Agitation wurden mit der Richmond Agitation-Sedation Scale (RASS) erfasst [34]. Die RASS ordnet psychomotorischen Zuständen einen Zahlenwert zu. Patienten, die äußerlich aufmerksam und ruhig wirken, haben einen RASS-Wert von null. Agitierte Patienten erhalten positive Werte (RASS +1 bis +4), somnolente oder sogar komatöse Patienten negative RASS-Werte (RASS −1 bis −5). Zum Ausschluss von Schmerzen wurde im Rahmen der klinischen Routine die Behavioral Pain Scale für intubierte Patienten (BPS) bzw. nichtintubierte Patienten (NI-BPS) verwendet [5, 26].

### Datenverarbeitung und Analyse

Für die demografischen Daten wurden stetige Variablen mit dem Mann-Whitney-U-Test verglichen, dichotome Variablen mit dem Chi-Quadrat-Test. Ein *p* < 0,05 wurde als statistisch signifikant gewertet. Für CAM-ICU und CAM-IMC sowie für „auffällige Vigilanz“ und „Desorientierung“ wurden Sensitivität, Spezifität, positiver prädiktiver Wert (PPW) und negativer prädiktiver Wert (NPV) berechnet. Unterschiede zwischen den drei Tests wurden mit dem Fisher-Exact-Test bestimmt. Die Analysen wurden mit Microsoft® Excel for Mac (Version 16.45, Microsoft Deutschland GmbH, München, Deutschland), SPSS (Version 26, IBM Corp.©, Ehningen, Deutschland) und Prism 5.0 (Graphpad Software Inc., La Jolla, CA, USA) durchgeführt.

## Ergebnisse

Insgesamt 86 gepaarte Untersuchungen fanden bei 50 extubierten Patienten statt. Ein Patient wurde ausgeschlossen, da er infolge eines prolongierten Komas als Folge eines Schlaganfalls während des Untersuchungszeitraums nie hinsichtlich eines Delirs untersuchbar war, und ein weiterer, weil er während des gesamten Studienzeitraums nur intubiert und somit die Orientierung nie zu testen war. Die demografischen Patientendaten sind in Tab. 1 zusammengefasst.

**Table 1:** 

		***N*** **=**	**Anzahl**	**%**
Geschlecht	m/w	50	35/15	70/30
		***N*** **=**	**Median**	**IQR**
Alter	Jahre	50	73	[67–79]
Größe	cm	50	172	[163–176]
Gewicht	kg	50	80	[71–95]
Mini-Cog	Punkte	50	3	[2–5]
Simplified Acute Physiology Score (SAPS II) Aufnahme	Punkte	50	32	[26–38]
Euro-Score	Punkte	50	7,5	[2,6–14,3]
Hämoglobin	g/dl	50	13,5	[12,3–14,5]
Thrombozyten	1000/µl	50	224	[189–276]
S‑Kreatinin	mg/dl	50	1,0	[0,9–1,4]
Bilirubin	mg/dl	50	0,6	[0,4–0,9]
Leukozyten	10^9^/l	50	7,6	[6,5–9,2]
C-reaktives Protein (CRP)	mg/dl	50	0,5	[0,0–2,0]

**Figure 2:**
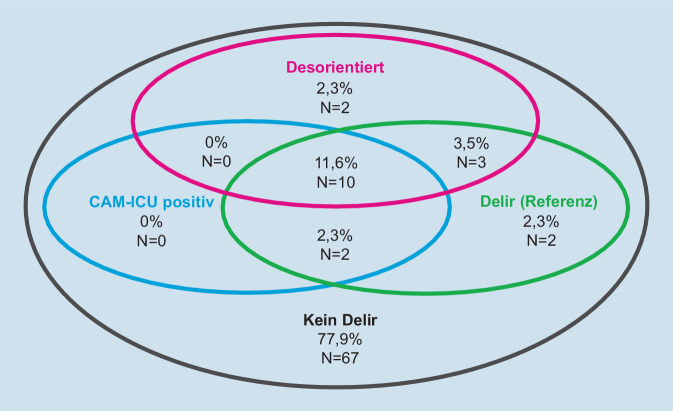

Ein Delir fand sich bei 19,8 % (*N* = 17) der Untersuchungen. Die Abb. 2 verdeutlicht die Zusammenhänge von Delir, CAM-ICU(Confusion Assessment Method for Intensive Care Units)-Befunden und Desorientierung zum Zeitpunkt der Untersuchung. In zwei Fällen lag trotz Delir keine Desorientierung vor. Umgekehrt gab es zwei Fälle von Desorientierung, in denen kein Delir nachweisbar. Diese beiden letztgenannten Patienten waren die einzigen, die nur zu einer einzigen Qualität nicht orientiert waren; eine konnte ihr Alter nicht nennen, der andere das Jahr nicht benennen. Beide entwickelten auch an den Folgetagen kein Delir. Auffallend war bei beiden ein niedriger Mini-Cog-Score (null und zwei Punkte) als Zeichen einer möglichen präoperativen kognitiven Einschränkung.

Die CAM-ICU fand nur in 14 % (*N* = 12) der Untersuchungen ein Delir. Die meisten dieser CAM-ICU-positiven Patienten waren auch desorientiert (*N* = 10), zwei waren hingegen voll orientiert. Falsch-positive Befunde kamen mit der CAM-ICU nie vor, ebenso fand sich bei CAM-ICU-negativen Befunden nie eine Desorientierung. Zwei desorientierte Patienten absolvierten die CAM-ICU fehlerfrei. Die Tab. 2 zeigt, dass sich für die CAM-ICU eine Sensitivität von 71 % (44–90 %) und eine Spezifität von 100 % (95–100 %) errechnete.

**Table 2:** 

	Sensitivität	Spezifität	PPV	NPV
CAM-IMC	88 % (64–99 %)	100 % (95–100 %)	100 % (78–100 %)	97 % (90–100 %)
Desorientierung	77 % (50–93 %)	97 % (89–100 %)	87 % (60–98 %)	94 % (86–98 %)
CAM-ICU	71 % (44–90 %)	100 % (95–100 %)	100 % (74–100 %)	93 % (85–98 %)
RASS ≠ 0	53 % (28–77 %)	91 % (82–96 %)	56 % (30–80 %)	90 % (81–95 %)

*CAM-IMC* Confusion Assessment Method für Intermediate Care, *CAM-ICU* Confusion Assessment Method for Intensive Care Units, *RASS* Richmond Agitation-Sedation Scale, *PPV* positiver prädiktiver Wert, *NPV* negativer prädiktiver Wert

Eine „auffällige Vigilanz“, also entweder agitiertes Verhalten (RASS > 0) oder Somnolenz (RASS < 0), fand sich bei jeweils acht Untersuchungen bei Patienten mit Delir. Acht Patienten mit Delir wirkten äußerlich aufmerksam und ruhig (RASS = 0). Die Tab. 2 zeigt, dass das Merkmal „auffällige Vigilanz“ (RASS ≠ 0) hinsichtlich der Detektion eines Delirs eine Sensitivität von nur 53 % (28–77 %) hatte, wohingegen die Spezifität immerhin bei 91 % (82–96 %) lag.

Acht Patienten hatten eine Aufmerksamkeitsstörung, wirkten aber äußerlich ruhig und aufmerksam (RASS = 0). Bei diesen ist laut CAM-ICU-Algorithmus die Überprüfung einer Denkstörung erforderlich. Nur in drei der acht Fälle war diese vorhanden, die CAM-ICU also positiv. Von den fünf Patienten ohne Denkstörung waren drei desorientiert, zwei nicht. Umgekehrt wurde bei keinem voll orientierten Patienten eine Denkstörung nachgewiesen. Dies führte zu der Entscheidung, für die Berechnung der CAM-IMC die Denkstörung ersatzlos gegen die Desorientierung auszutauschen (Abb. 1).

Desorientierung, als alleiniges Delir-Merkmal verwendet, identifizierte in 15,1 % (*N* = 13) zutreffend ein Delir. Vier Patienten mit Delir waren hingegen für alle getesteten Qualitäten orientiert (Abb. 1). Da die Desorientierung eine signifikant höhere Sensitivität, die CAM-ICU hingegen eine höhere Spezifität zeigte, wurde die CAM-ICU mit dem Merkmal „Desorientierung“ kombiniert. Diese Variante wurde als „CAM-IMC“ bezeichnet (Abb. 1). Für die CAM-IMC errechnete sich eine Sensitivität von 88 % (95%-KI: 64–99 %) und eine Spezifität von 100 % (95–100 %; Tab. 2).

**Figure 3:**
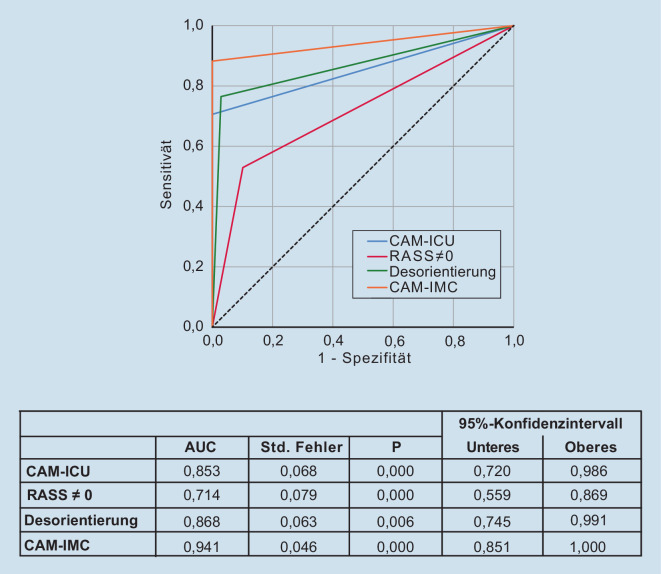

Die Abb. 3 zeigt die Kurven der „receiver operating characteristics“ (ROC-Kurven) für die einzelnen Delir-Merkmale bzw. Delir-Tests. Sie verdeutlicht, dass die ROC-Kurve der „Bewusstseinsveränderung“ (RASS ≠ 0) unter allen anderen Kurven lag und damit den ungünstigsten Vorhersagewert vorzuweisen hat. CAM-ICU und „Desorientierung“ weisen jeweils eine vergleichbare „area under the curve“ (AUC) auf. Die CAM-IMC weist mit der höchsten ROC-AUC die günstigsten Testgütekriterien auf.

## Diskussion

Diese Arbeit wurde mit dem Ziel durchgeführt, die Wertigkeit der Desorientierung zur Erfassung eines Delirs festzustellen. Gleichzeitig sollte ermittelt werden, ob sich die Testgütekriterien der CAM-ICU durch das Merkmal „Desorientierung“ („CAM-IMC“) verbessern lassen. Es fand sich, dass alleine das Merkmal „Desorientierung“ bei extubierten Patienten ein sensitiveres Merkmal als die CAM-ICU ist, um ein mögliches Delir zu entdecken. Eine allgemeine „psychische Veränderung“ allein hatte eine vergleichsweise niedrige Sensitivität für das postoperative Delir. Die CAM-IMC – eine Kombination der CAM-ICU mit dem Merkmal „Desorientierung“ – hatte insgesamt die günstigsten Testgütekriterien unter den untersuchten Delir-Tests. Es fand sich eine höhere Sensitivität als bei der CAM-ICU bei gleichzeitig sehr hoher Spezifität.

Diese Arbeit bestätigt eine Studie von Bellelli et al., in der die Orientierungsstörung sich als ein sensitives Merkmal für die Vorhandensein eines möglichen Delirs bei eher niedriger Spezifität fand [2]. Auch in der vorliegenden Arbeit fanden sich einige Patienten, die zwar desorientiert, aber nicht delirant waren. Zwei Patienten, die lediglich zeitlich desorientiert waren, entwickelten nie ein Delir, hatten aber Hinweise auf mögliche präoperative kognitive Einschränkungen. Unsere Befunde passen demnach auch zu der schon 1956 publizierten Beobachtung, dass die „zeitliche Orientiertheit“ die vulnerabelste Qualität der Orientierung sei, und dass es „partielle Verlaufsformen“ gibt, die unter Umständen nicht das Vollbild eines Delirs erreichen [21]. Somit kommt der Prüfung der Desorientierung bei nichtintubierten Patienten eine herausragende Bedeutung zu.

Andere Arbeiten bei geriatrischen und neurochirurgischen Patienten schlussfolgerten, dass eine Bewusstseinseinschränkung (erfasst als RASS ≠ 0) ein geeignetes Screening-Instrument für ein Delir sei [6, 16, 25, 28]. Das mag bei Patienten zutreffen, bei denen keine beeinträchtigte Kognition und Wachheit zu erwarten sind. Bei Patienten mit ausgeprägten Traumata, Infektionen und großen operativen Eingriffen scheint die RASS als alleiniges Screening-Instrument allerdings weit weniger geeignet als die Desorientierung. Eine jüngst publizierte Arbeit untersuchte die genannten Delir-Merkmale in Zusammenhang mit der Früherkennung einer Sepsis durch den „qSOFA“ [22]. Die dem qSOFA zugrunde liegenden Sepsis-3-Kriterien sehen für die Erfassung der „Bewusstseinsveränderung“ die Glasgow Coma Scale (GCS) vor [35]. Für die genannte Arbeit wurde die GCS entweder durch „4 Fragen zur Orientierung“ (qSOFA_Ox4_) oder „RASS ≠ 0“ (qSOFA_RASS_) ersetzt [22]. Für beide qSOFA-Varianten fanden sich eine vergleichsweise niedrige Sensitivität hinsichtlich des Vorliegens einer Sepsis (52,3 % bzw. 57,1 %) und keine wesentlichen Unterschiede zwischen den bei beiden Parametern. Hervorzuheben ist, dass – wie in vorangegangen Arbeiten – auch in unserer Arbeit ein RASS = 0 ein Delir keineswegs ausschloss, was die Vigilanz als alleiniges Instrument bei der Delir-Einschätzung rein postoperativer Patienten als ungeeignet erscheinen lässt [11, 13].

Wegen der vergleichsweisen niedrigen Spezifität der Desorientierung ist die zusätzliche Testung eines weiteren Merkmals mit hoher Spezifität notwendig. Auch dem Ausschluss eines Delirs kommt eine hohe Bedeutung zu. So dürfen z. B. Schmerzen keinesfalls übersehen (bzw. fehlinterpretiert) werden, weil Patienten mit Schmerzen fälschlicherweise als delirant eingeschätzt werden können [19]. Darüber hinaus ist Schmerz selbst ein Risikofaktor für ein Delir [19]. Der wesentliche Vorteil der CAM-ICU liegt in ihrer hohen Spezifität bei postoperativen Patienten [12, 23]. Kein Patient, der laut Referenzuntersucher frei von Delir war, wurde laut CAM-ICU als falsch-positiv eingestuft. Dies ist vor allem die Folge der hohen Gewichtung der „Aufmerksamkeitsstörung“ bei der CAM-ICU, ohne deren Vorhandensein der Test nicht positiv wird.

Für das Testen der Aufmerksamkeitsstörung wurde für diese Arbeit der Vigilance-„A“-Test („ANANASBAUM“) auch im Rahmen des vom Referenzuntersucher eingesetzten 4AT verwendet. Die Aufmerksamkeitsstörung wird beim 4AT üblicherweise durch „Aufsagenlassen von sieben Monaten rückwärts“ überprüft („months of the year backwards“, MOTYB). Dies geschah, weil der „ANANASBAUM“ durch die CAM-ICU auf vielen deutschsprachigen Intensivstationen weit verbreitet ist [20, 33]. Zwischen MOTYB und Vigilance-„A“-Test fanden sich keine signifikanten Differenzen hinsichtlich der Sensitivität, bei etwas höhere Spezifität des MOTYB [1].

Allerdings waren drei Patienten ohne Aufmerksamkeitsstörungen desorientiert und delirant im Sinne der vom Referenzuntersucher verwendeten Delir-Kriterien. Die höhere Rate deliranter Befunde mit dem 4AT im Vergleich zur CAM-ICU erklärt sich unter anderem dadurch, dass im 4AT der Desorientierung sowie dem fluktuierenden Verlauf eine besondere hohe Bedeutung zukommt. So erlangen beispielsweise erstmals desorientierte Patienten allein durch das Merkmal „Desorientierung“ und das automatisch positive Merkmal „fluktuierende Symptomatik“ selbst bei Vorhandensein der Desorientierung zu nur einer Qualität bereits fünf Punkte. Ab vier Punkten ist nach den Kriterien des 4AT – also auch in Abwesenheit einer Aufmerksamkeitsstörung – ein „Delir möglich“ [2, 32].

Der hohen Spezifität der CAM-ICU bei nichtintubierten Patienten steht die vergleichsweise niedrige Sensitivität gegenüber (Tab. 2). Aus diesem Grund wurde die CAM-IMC zusammengestellt (Abb. 1). Zugunsten des Merkmals „Desorientierung“ wurde bei der CAM-IMC auf das Merkmal „Denkstörung“ verzichtet, da bei keinem voll orientierten Patienten je eine Denkstörung nachgewiesen wurde. Auf diese Weise ließ sich die vergleichsweise hohe Sensitivität des Merkmals „Desorientierung“ kombinieren mit der hohen Spezifität, die mit dem Testen der Aufmerksamkeitsstörung einhergeht. Weitere Arbeiten müssen zeigen, ob sich die hier ermittelten günstigen Testgütekriterien sich in einer größer angelegten Validierungsstudie bestätigen lassen.

### Limitationen

Diese Arbeit hat einige Limitationen: Erstens handelt es sich bei der vorliegenden Arbeit um eine retrograde Analyse mit limitierter Fallzahl. Die Studie war für eine andere Fragestellung geplant und durchgeführt worden und entsprechend dafür die Fallzahl kalkuliert. Zweitens ist die vorliegende Arbeit eine sekundäre Analyse, für die ausschließlich Messungen bei extubierten Patienten ausgewertet wurden, sodass sich hierdurch eine niedrigere Fallzahl ergab. Drittens ist die CAM-IMC auf der Grundlage dieses limitierten Datensatzes zusammengestellt worden. Es handelt sich nicht um eine Validierungsstudie. Eine größer angelegte prospektive Studie ist daher notwendig, um die klinische Wertigkeit der CAM-IMC und ihrer Testgütekriterien zu verifizieren.

## Schlussfolgerung

Die Erfassung der „Desorientierung“ und deren Einbeziehung führte zu einer signifikant höheren Anzahl von erkannten Delir-Patienten, als wenn die CAM-ICU als alleiniges Werkzeug genutzt worden wäre. Anderseits hat sich gezeigt, dass nicht alle desorientierten Patienten alle Kriterien für ein Delir erfüllen, noch dass diese zu einem späteren Zeitpunkt delirant werden. Die Kombination der CAM-ICU mit der „Desorientierung“ als CAM-IMC verstärkte die Sensitivität und erhöhte sie noch über die Werte der Einzeltests hinaus bei unverändert hoher Spezifität. Diese Arbeit unterstreicht die Wertigkeit des Merkmals „Desorientierung“ und erklärt zweifellos einige diskrepante Beurteilungen schwierig einzuschätzender Patienten in der täglichen Praxis. Diese Studie bestätigt überdies die Notwendigkeit präoperativer Kognitionstests, insbesondere der verschiedenen Qualitäten der Orientierung, um später das Merkmal „fluktuierender Verlauf“ zutreffend einschätzen zu können. Weitere Forschung sollte hinsichtlich der Wertung einzelner Delir-Kriterien angestrengt werden sowie eine prospektive Validierung der CAM-IMC vorgenommen werden.
